# Investigation of SARS-CoV-2 Variants and Their Effect on SARS-CoV-2 Monoclonal Antibodies, Convalescent and Vaccine Plasma by a Novel Web Tool

**DOI:** 10.3390/diagnostics12112869

**Published:** 2022-11-19

**Authors:** Ayse Arikan, Murat Sayan

**Affiliations:** 1 Department of Medical Microbiology and Clinical Microbiology, Faculty of Medicine, Near East University, Nicosia 99138, Northern Cyprus, Turkey; 2 DESAM Research Institute, Near East University, Nicosia 99138, Northern Cyprus, Turkey; 3 Department of Medical Microbiology and Clinical Microbiology, Faculty of Medicine, Kyrenia University, Kyrenia 99320, Northern Cyprus, Turkey; 4 PCR Unit, Research and Education Hospital, Kocaeli University, Izmit 41380, Kocaeli, Turkey

**Keywords:** SARS-CoV-2 variant, therapeutic, vaccine, bioinformatics

## Abstract

(1) Background: SARS-CoV-2 variants possess specific mutations throughout their genome; however, the effect of these mutations on pathogenesis is little known. The study aimed to identify SARS-CoV-2 variants and their susceptibility rate against monoclonal antibodies, convalescent, and vaccine plasma. (2) Methods: Strains isolated from COVID-19 cases in Turkey in April and September 2021 were involved. Illuma Nextera XT was processed for NGS, followed by virtual phenotyping (Coronavirus Antiviral and Resistance Database (CoV-RDB) by Stanford University). (3) Results: Among 211 strains, 79% were SARS-CoV-2 variants. B.1.1.7 (Alpha) was the most dominant, followed by B.1.617.2 (Delta), B.1.351 (Beta), and B.1.525 (Eta). Alpha and Delta were less susceptible to Etesevimab—Sotrovimab and Bamlanivimab—Etesevimab, respectively. Reduced efficacy was observed for convalescent plasma in Beta and Delta; AstraZeneca, Comirnaty plus AstraZeneca in Alpha; Comirnaty, Moderna, Novovax in Beta; Comirnaty in Delta. (4) Conclusion: CoV-RDB analysis is an efficient, rapid, and helpful web tool for SARS-CoV-2 variant detection and susceptibility analysis.

## 1. Introduction

Novel coronavirus disease (COVID-19), caused by severe acute respiratory syndrome coronavirus 2 (SARS-CoV-2), has become a global threat since it first emerged in December 2019 [1]. The new coronavirus is contagious and has spread more efficiently worldwide, causing detrimental results, mainly lung diseases, especially in hospitalized cases [2]. By 8 November 2022, more than 620 million confirmed cases had been reported, with over 6.5 million deaths [3]. SARS-CoV-2, belonging to the *Coronaviridae* family, is a single-stranded, positive-sense RNA virus that may exhibit high genetic diversity [4,5]. Due to the high substitution rate (8 × 10^−4^ subs per site per year) of SARS-CoV-2, variants originate from nucleotide mutations [5]. The dissemination and evolution of new SARS-CoV-2 variants should be identified to understand better these genetic changes’ effect on the virus’s transmission rate and its impact on vaccines and therapeutics [6,7,8]. Different approaches have been developed to help physicians improve patient management and reduce mortality rates since the first emergence of the variants of SARS-CoV-2 [9,10,11]. Machine learning has become a more valuable and popular tool to predict COVID-19 cases in the early stages and identify patients with high mortality risk instead of using conventional techniques [10,11].

Genomic characterization of SARS-CoV-2 strains isolated from COVID-19 cases indicates decreased neutralization efficacy of anti-SARS-CoV-2 monoclonal antibodies (mAb), convalescent plasma (CP), and vaccine-derived plasma (VP) against these variants [12,13,14]. Currently, Omicron (B.1.1.529) lineages continue to be the circulating dominant variants of concern (VOCs) as they have been reported to be more contagious, despite lower disease severity than other lineages [15,16,17]. Recent information on changes in COVID-19 epidemiology, clinical disease outcomes, and efficacy of diagnostics, vaccines, and therapeutics due to viral genome diversity is critically important to achieving control of the global pandemic. Characterizing these variants by whole genome sequencing and virtual phenotyping methods helps assist genomic surveillance [18,19]. Stanford Coronavirus Antiviral and Resistance Database (CoV-RDB) by Stanford University has been developed to assess viral sequence alignments by utilizing predetermined consensus SARS-CoV-2 sequences not only for variant detection but also the susceptibility of these variants to mAbs, CP, and vaccine plasma. The CoV-RDB has been used to track variants since August 2020 and showed a high agreement with the gold standard phylogenetic analysis [20,21]. The CoV-RDB includes in vitro, animal model, and clinical trial data for candidate anti-SARS-CoV-2 compounds and experimental results on viral gene diversity, lineage, and susceptibility to mAbs, CP, and vaccine plasma at https://covdb.stanford.edu/ (accessed on 7 December 2021). The database is freely accessible, easy to use, and rapid. The system accepts the sequences as plain text if only one sequence is analyzed and the FASTA format if more than one sequence is submitted. The upper limit is currently 100 sequences containing ~30,000 nucleotides per sequence. The input sequences are aligned to the reference sequence Wuhan-Hu-1 (NC_045512.2), and the report provides information on lineage/variant and mutations, a quality assessment, drug resistance comments, and a susceptibility summary [22].

Vaccination is the leading solution to control the COVID-19 pandemic; several companies have developed different technologies against SARS-CoV-2. Several vaccine technologies, including m RNA, viral vector, inactive, recombinant, subunit protein, and combined vaccines, have been used to prevent severe disease and hospitalization due to COVID-19 [23]. Different technologies have been shown to have separate administration doses, age groups, and efficacy for achieving immunity [24]. However, due to the emergence of new variants, studies need to be continued to evaluate the mechanism of action, efficacy, safety, and the correct use of vaccine doses of the most widely used COVID-19 vaccines.

The diversity of SARS-CoV-2 strains in many countries provides epidemiological data for SARS-CoV-2 surveillance and information for identifying target sites for vaccine and drug development. Therefore, the novelty of the study was: (i) to identify SARS-CoV-2 mutations and variants in Turkish COVID-19 cases, (ii) to evaluate the susceptibility rate of identified variants to anti-SARS-CoV-2 mAb drugs, (iii) to evaluate the potential neutralizing efficacy of CP obtained from naturally infected cases and VP from vaccinated individuals against these variants with a newly designed web tool for SARS-CoV-2 diagnosis. To the best of our knowledge, the study will be the first that examines SARS-CoV-2 variants and their effects on pathogenesis using the virtual phenotyping method.

## 2. Materials and Method

### 2.1. Study Patients

Two-hundred-and-eleven SARS-CoV-2 variant strains isolated from SARS-CoV-2 infected cases in Kocaeli, Istanbul, and Ankara, Turkey, in April 2021 and September 2021, which were predicted as SARS-CoV-2 variations by two different SARS-CoV-2 specific PCR variant kits, were included in the study.

### 2.2. SARS-CoV-2 Viral RNA Extraction and Amplification

To isolate SARS-CoV-2 RNA from the nasal/oropharyngeal specimens, the fully automated rotary nucleic acid magnetic particle extraction system Auto Extractor GeneRotex96 (Tianlong Science and Technology Co., Xi’an City, China) was used. A standard PCR kit with a double gene target (BioSpeedy, Bioeksen Inc., Istanbul, Turkey) was used for the viral detection.

### 2.3. SARS-CoV-2 Variant Screening PCR

Two variant-specific screening PCR kits (BioSpeedy SARS-CoV-2 N501Y/variant plus kit, Bioeksen Inc., Istanbul, Turkey, and Diagnovital SARS-CoV-2 N501Y, delHV69-70, E484K mutation detection kit, RTA Laboratories Inc., Istanbul, Turkey) were used to predict potential SARS-CoV-2 variants.

### 2.4. SARS-CoV-2 Spike Gene Sequencing, Variants and Patterns

Variant predicted strains were analyzed by next-generation sequencing (NGS). A NucleoFast 96 PCR kit (Macherey-Nagel GmbH, Dueren, Germany) and quantitation in spectrophotometry (Nanodrop N1000, Thermo Fisher Inc., Wilmington, DE, USA) was used for the purification of SARS-CoV-2 real-time PCR products. The nucleic acid concentration was standardized to 0.2 ng/uL for each sample and was processed by NexteraXT (Illumina Inc., San Diego, CA, USA) for NGS.

*Spike* glycoprotein receptor binding domains between 21,709–23,193 bps of SARS-CoV-2 Wuhan Hu-1 isolate with MN908947.3 GenBank accession number were targeted. The primers zone (~1500 bp) between 118F–1652R was sequenced, and R: 5′- acacctgtgcctgttaaacca—3′ and F: 5′gacaaagttttcagatcctcagttttaca—3′. were used as the primer pairs for the sequence [21,25]. NGS sequencing was carried out on the Miseq (Illumina Inc., San Diego, CA, USA) platform, and the following conditions were processed for the *Spike* NGS PCR amplification: 45 °C for 10 min, 95 °C for 2 min, 95 °C for 10 s, 57 °C for 30 s, and 72 C for 30 s for 40 cycles. The Miseq Reporter, based on BWA software (http://bio-bwa.sourceforge.net/ (accessed on 7 December 2021)), aligned the resulting sequences.

The nucleotide sequences of the SARS-CoV-2 strains were identified using the CoV-RDB by Stanford University tool (https://covdb.stanford.edu/sierra/sars2/by-sequences/ (accessed on 7 December 2021)). The initial analysis was performed in June 2021 with 143 strains, then in July 2021 with 25 strains, and finally at the end of September 2021 with 43 additional strains. The strains analyzed in June and July 2021 were reviewed in September 2021 as we noticed that Johnson & Johnson (AD26.COV2.S) had been added to the database.

### 2.5. SARS-CoV-2 mAbs Susceptibility

The SARS-CoV-2 mutation patterns and the understanding of these mutations’ neutralizing effectivity against therapeutics and antibodies came from naturally infected and vaccinated individuals (hereafter referred to as vaccine-elicited susceptibility) by the virtual phenotyping method.

The sensitivity rate of the identified strains to mAbs which either have emergency use authorization (EMU) or are under clinical trial [26] were: Bamlanivimab (BAM) (LY-CoV555) (Eli Lilly and Company, Indianapolis, ID, USA), Etesevimab (ETE) (LY-CoV016) (Eli Lilly and Company, Indianapolis, ID, USA), Casirivimab (CAS) (Regeneron Pharmaceuticals, NY, USA), Imdevimab (IMD) (REGN10987) (Regeneron Pharmaceuticals, NY, USA), VIR-7831 aka Sotrovimab (SOT) (VIR-7831) (GlaxoSmithKline and Vir Biotechnology, NC, USA), Cilgavimab (CIL) (COV2-2130 or AZD 1061) (AstraZeneca/Vanderbitt, Cambridge, UK), Tixagevimab (TIX) (COV2-2196 or AZD8955) (AstraZeneca, Cambridge, UK), C135 (Rocketefeller University, Roche, NY, USA), C144 (Rocketefeller University, Roche, NY, USA), BRII-196 (BRII Biosciences, Durham, NC, USA), BRII-198 (NCT-04479644) (BRII Biosciences, Durham, USA), and antibody cocktails involving BAM plus ETE (Eli Lilly and Company, Indianapolis, ID, USA), CAS plus IMD (Regeneron Pharmaceuticals, New York, NY, USA), CIL plus TIX (AstraZeneca, Cambridge, UK), C135 plus C144 and BRII-196 plus BRII 198 (Bri Biosciences, Durham, NC, USA) with SARS-CoV-2 mAb trackers [27,28,29,30] and the susceptibility levels were interpreted as ‘<3-fold: susceptible; 3–9-fold: intermediate; ≥10-fold: resistance’ according to the CoV-RDB recommendations [26].

### 2.6. CP and Vaccine-Elicited Plasma Susceptibility

Mutations which contributed to evading the immune response were evaluated among individuals with natural and vaccine-induced immunity. The susceptibility levels for both categories were considered median fold and interpreted as ‘<3-fold: susceptible; 3–9-fold: intermediate; ≥10-fold: resistance’ according to the CoV-RDB recommendations [22,26].

The COVID-19 vaccines involved in the study were categorized as [31,32] (i) messenger (m) RNA vaccine involving: Comirnaty (previously named BionTech, Pfizer) (BNT 162b2) (BioNTech, Fosun Pharma, Pfizer, NY, USA) and *Spike*vax (previously named COVID-19 Vaccine Moderna) (mRNA-1273) (ModernaTX Inc., Cambridge, USA); (ii) viral vector vaccine involving: AstraZeneca (AZD1222) (Pharmaceutical Biotechnology, Cambridge, UK), Sputnik V (Gamaleya Research Institute of Epidemiology and Microbiology, Moscow, Russia), Johnson & Johnson (AD26.COV2.S) (Janssen Pharmaceutical Companies, Horsham, PA, USA); (iii) inactivated vaccine involving: Bharat Biotech (BBV154) (Bharat Biotech, Telangana, India), Sinopharm (BIBP COVID-19) (Sinopharm’s Beijing Institute of Biological Products, Beijing, China), Covaxin (BBV152) (Bharat Biotech, Telangana, India), and CoronaVac (SinoVac-CoronaVac) (Sinovac Biotech, Beijing, China); (iv) recombinant vaccine involving: Novovax (NVX-CoV2373) (Novovax, Maryland, US and the Coalition for Epidemic Preparedness Innovations, London, England), Medigen (MVC-COV1901) (Medigen Vaccine Biologics Corporation, Taipei, Taiwan); and (v) combination vaccine involving: BionTech, Pfizer and AstraZeneca [26,33,34].

### 2.7. Ethical Approval

The study was retrospectively conducted and approved by the Near East University (NEU) Scientific Research Ethics Committee (decision number 1383 NEU/2021/93).

## 3. Results

### 3.1. Spike Variants and Mutations

Due to spike gene NGS analysis of the 211 SARS-CoV-2 variant suspected strains, 72% (n = 152) were confirmed as new forms of SARS-CoV-2 while 28% (n = 59) were identified as wild type (WT). In the time interval, Alpha (B.1.1.7), Beta (B.1.351), Delta (B.1.617.2), and Eta (B.1.525) variants were reported throughout the three largest cities of Turkey. The Alpha (B.1.1.7) variant was the most predominant variant with a rate of 88% (n = 134), followed by B.1.617.2 (Delta) with a rate of 7% (n = 10). Circulating Beta (B.1.351) and Eta (B.1.525) variants were noted with a rate of 4% (n = 7) and 1% (n = 1), respectively.

Among the Alpha variants, the most common mutation pattern was ∆69–70, ∆144, N501Y at 78% (n = 105), while other Alpha mutations involving N501Y were also significant at 22% (n = 29). The Beta (B.1.351) variant was obtained from seven COVID-19 cases, and D80A, D215G, ∆241–243, K417N, E484K, and N501Y was the only pattern determined. Among the Delta (B.1.617.2) strains, T95I, G142D, ∆156–157, R158G, L452R, and T478K were described as the dominant patterns (n = 3, 30%). T478K (n = 2, 20%); G142D, ∆156–157, R158G, L452R, T478K (n = 1, 10%); G142D, ∆156–157, R158G, A222V, L452R, T478K (n = 1, 10%); G142D, ∆156–157, R158G, N440T, L452R (n = 1, 10%); L452R, N501Y (n = 1, 10%); and A222V (n = 1, 10%) were the other mutations determined as Delta variants in Turkey. The mutation pattern A67V, ∆69–70, ∆144, E484K was described in one case and identified as Eta (B.1.525) in this study.

The prevalence of SARS-CoV-2 lineage and variants identified in the study patients is given in Table 1.

### 3.2. MAbs Susceptibility

The susceptibility rate of the SARS-CoV-2 mAb drugs used for COVID-19 treatment was evaluated by the genomic characterization of SARS-CoV-2 with the CoV-RDB. Our findings revealed that the majority of mAbs tested in the study exhibited suitable neutralizing activities against the Alpha variant, except for the ETE mAb (n = 111, 83%), which showed diminished susceptibility to B.1.1.7. On the other hand, we reported that the mAb cocktail of ETE with BAM promoted the antibodies’ efficacy against the Alpha variants. Another critical finding was noted for the SOT mAb. In 111 (83%) Alpha variants the potential therapeutic effect of the SOT mAb was decreased.

The study only obtained the antibody effect in Beta variants for the BAM and ETE mAbs. According to the results, Beta variants (n = 7, 100%) were identified as SARS-CoV-2 strains resistant to the ETE mAb.

Resistance to BAM and ETE mAbs was reported in 60% and 10% of the cases of Delta strains, respectively. We observed that the efficacy increased with BAM and ETE antibody cocktail administration. It was also noted that the effectiveness of CAS (40%) and a CAS plus IMD (10%) mAb cocktail exhibited less susceptibility to Delta variants.

The susceptibility rate of all SARS-CoV-2 lineages to mAbs is represented in Table 2.

### 3.3. CP Susceptibility

While all the Alpha strains (n = 134, 100%) among the whole SARS-CoV-2 variant strains isolated from COVID-19 cases in Turkey were reported as susceptible, the Beta strains (n = 7, 100%) isolated from the study patients were less sensitive to neutralization by convalescent plasma (CP). For the Delta variants, it was reported that only the strains with the mutation patterns (T95I, G142D, ∆156–157, R158G, L452R, T478K), (G142D, ∆156–157, R158G, A222V, L452R), and (T478K) exhibited reduced susceptibility to neutralization by CP. The susceptibility rate to CP in the determined SARS-CoV-2 variants is given in Table 3.

### 3.4. VP Plasma Susceptibility

We noted that most of the COVID-19 vaccines evaluated here were effective against SARS-CoV-2 Alpha variants, except for AstraZeneca and the Pfizer BioNTech plus AstraZeneca vaccine combination. A reduced susceptibility to both vaccines was determined in 85% of the cases infected with SARS-CoV-2 Alpha variants. In addition, diminished susceptibility-associated strains were also obtained from the B.1.351 lineage for Pfizer BioNTech, Moderna, and Novovax, with rates of 71%, 29%, and 14%, respectively. Moreover, our results determined that the Pfizer BioNTech vaccine was less effective against 10% of Delta variants. On the other hand, the potential effect of BioNTech, Pfizer, and Moderna vaccines against Eta variants was noted in the study. Table 4 shows the susceptibility rate of the SARS-CoV-2 strain to COVID-19 vaccines.

Due to the continual updates to the variant databases, we re-identified the sequences we defined in June and July 2021. Except for adding a new vaccine to the analysis, our results were similar.

## 4. Discussion

Since its first emergence in December 2019, SARS-CoV-2’s genomic diversity and genomic epidemiology have been investigated worldwide to understand the rapid spread of new agents better. The consequential impact on the severity of the infection and whether the newly emerging variants have a diminishing effect on the benefits of vaccines and treatment alternatives are being evaluated to guide the management of the ongoing COVID-19 pandemic [35,36,37]. This study identified SARS-CoV-2 variant strains (72%) isolated from Turkish patients by NGS, followed by the virtual phenotyping method. In Turkey, B.1.1.7 (Alpha) was the most dominant variant, followed by B.1.617.2 (Delta), B.1.351 (Beta), and B.1.525 (Eta). The susceptibility patterns of these variants against mAbs and vaccines revealed that Alpha and Delta were less susceptible to Etesevimab—Sotrovimab and Bamlanivimab—Etesevimab, respectively. Additionally, reduced efficacy was observed for convalescent plasma in Beta and Delta; AstraZeneca and Comirnaty plus AstraZeneca in Alpha; Comirnaty, Moderna, and Novovax in Beta; and Comirnaty in Delta.

In the current study, we chose to use the CoV-RDB because it provides SARS-CoV-2 variants and their associated mutations in a single analysis and provides sensitivity Measurements for these variants to mAbs, CP, and vaccine plasma. Our findings revealed three lineages from the VOC category at that period involving B.1.1.7 (Alpha), B.1.351 (Beta), and B.1.617 (Delta). Of these lineages, the B.1.1.7 (Alpha) variant was predominant in Turkey (88%) until September 2021. Globally, the Alpha variant was reported in 193 countries and was the dominant variant worldwide [38]. However, as the Delta variant (7%) was determined at a remarkable rate in the study in a given time interval, it could be predicted that Delta would be dominant in the future, since B.1.617 (Delta) has dominated the world since that date [39]. While the Delta variant continued to raise concerns, the newly identified B.1.1.529 (Omicron) variant confirmed its importance in the early detection of new variants by the large-scale analysis of SARS-CoV-2 *spike* gene mutations [40]. Our findings on SARS-CoV-2 variant distribution indicate that the detection of variants for continual surveillance is essential during the COVID-19 pandemic.

Furthermore, this research evaluated the viral neutralization activity of CP in the determined SARS-CoV-2 variants, as these strains can decrease neutralization by CP [5,12]. Similar studies on the susceptibility of SARS-CoV-2 variants to convalescent antibodies showed reduced neutralization activity [41,42]. Our findings showed that plasma containing antibodies produced from infected cases would be a helpful treatment alternative, especially against B.1.1.7 (Alpha) lineages, B.1.617.2 (Delta) lineage mutations with (G142D, ∆156–157, R158G, L452R, T478K), (L452R, N501Y), (T478K), and (A222V), and B.1.525 (Eta). The negative effect of mutations such as E484K on neutralization has been shown previously [43]. Here, we also noted that B.1.351 (Beta) and B.1.617 (Delta) lineages, including the (T95I, G142D, ∆156–157, R158G, L452R, T478K), (G142D, ∆156–157, R158G, A222V, L452R), and (T478K) mutations were associated with reduced susceptibility to CP; therefore, this treatment should be less preferred for individuals infected with one of these SARS-CoV-2 mutations.

Our genomic analysis also revealed the potency of the identified SARS-CoV-2 lineages to evade mAbs and vaccines. Recently, the Food and Drug Administration (FDA) authorized Bebtelovimab, SOT, BAM plus ETE, and CAS plus IMD (REGEN-CoV) mAb combinations for COVID-19 treatment [30]. The current study evaluated 11 mAbs and five mAb cocktails, three of which have been clinically approved, to investigate the potent effect of the determined variants against these therapeutic products. When administered alone, we found that the result of the monoclonal antibody ETE against the Alpha variant was not as effective as when administered with BAM (Table 2). We also found that the Alpha variants (83%) associated with ETE resistance became susceptible (98%) when using a BAM plus ETE cocktail treatment. Additionally, we reported a reduced susceptibility to SOT in a high percentage of Alpha variants (83%), suggesting that the use of the SOT antibody with any other compound may be a promising approach, as antibody cocktails have been described as more effective against SARS-CoV-2 variants in recent studies [7,44].

The Stanford database is updated frequently (~every month) [22,26]. Therefore, we reassessed the strains we identified and compared our results to avoid missing new updates. We wanted to ensure we obtained all the information because the databases were updated ~every month [22]. When we performed further analysis obtained from different time intervals, we realized that variant identification and their associated susceptibility data were the same except for VP due to adding a vaccine to the database. If we had not reanalyzed the sequences we analyzed at the beginning of the study, we would have missed new information. Therefore, it would be helpful and necessary to review the identified sequences if new sequences are added in the future. In this study, there was insufficient data on the susceptibility of many mAbs against Beta variants except for ETE. For the B.1.351 lineage, we noted resistance susceptibility associated with ETE variants (100%), indicating that ETE should be given with another compound to increase the efficacy of the antibody against the B.1.351 lineage. In addition, we reported that Delta variants have the potential to evade neutralization by antibodies such as BAM (60%) and ETE (20%) or to reduce the effect of some antibodies, including BAM plus ETE (10%), CAS (40%), and CAS plus IMD (10%). Emerging new variants and their adverse effects on treatment susceptibilities indicate the need to conduct sequencing analysis to combat the COVID-19 pandemic effectively.

Our most crucial solution in fighting the COVID-19 global pandemic is the rapid development and diversification of effective vaccines. Additionally, the current study provides data on vaccine efficacy against variants circulating in some parts of Turkey. Twelve different COVID-19 vaccines were evaluated: the m RNA vaccine, viral vector vaccine, inactivated vaccine, recombinant-based vaccine, and combination vaccine technologies. Here, we observed that, except for AstraZeneca and the BioNTech, Pfizer plus AstraZeneca vaccine combination, all COVID-19 vaccines were effective against B.1.1.7. Surprisingly, BioNTech and Pfizer were adequate; the efficacy was reduced with the AstraZeneca booster dose. As we observed with antibody cocktails, we could not obtain increased effectiveness by administering vaccine combinations. Although sufficient data on vaccine escape was obtained for B.1.1.7, more clinical data are needed for other emerging variants. We determined the impact of mRNA vaccines from BioNTech, Pfizer, and Moderna against B.1.351, B.1.617, B.1.525, and other missense mutations against the determined SARS-CoV-2 lineages. A reduction of vaccine-elicited plasma susceptibility was observed only against Beta variants. However, we did not determine any reduction for Delta, Eta, and other missense variants. In a study conducted in Qatar, Chemaitelly et al. showed the strong effect of the Moderna vaccine against B.1.1.7 and B.1.351 [45]. Similarly, Puranik et al. stated the robust protection of two mRNA vaccines against Alpha and Delta variants [46]. Although studies have evaluated the validity of COVID-19 vaccines against SARS-CoV-2 variants, further investigations on SARS-CoV-2 variants, their associated mutations, and the potential to evade immune responses and therapeutics should consistently be conducted.

In conclusion, as new variants of SARS-CoV-2 dominate and diversify, the efficacy of vaccines and therapeutics used to control the COVID-19 pandemic must be continuously investigated. The Stanford CoV-RDB is a promising approach for detecting variants and performing resistance analyses, as the newly developed web tool is rapid, practical, and freely accessible. Determining the impacts of different variants would guide physicians in managing cases, reducing mortality, and influencing public health strategies.

To point out the limitations of the study, we evaluated only the vaccines and monoclonal antibodies in the database during the specified time interval. This study did not assess vaccines and the mAbs that were subsequently approved for emergency use and newly added to the database. Additionally, the study included only COVID-19 cases detected between April 2021 and September 2022 in the Istanbul, Kocaeli and Ankara provinces. COVID-19 cases from different regions of Turkey should be added to show the genomic variations of SARS-CoV-2 in Turkey.

## Figures and Tables

**Table 1:** Prevalence of SARS-CoV-2 lineage and mutation patterns in the study patients.

SARS-Cov-2 Lineage	SARS-CoV-2 Mutation Pattern	CoV-RDB, n (%)
B.1.1.7/Alpha		134 (64)
	N501Y, ∆69–70, ∆144	105 (78)
	N501Y, ∆144	7 (5)
	N501Y	7 (5)
	N501Y, ∆69–70, S98F, ∆144	2 (1.4)
	N501Y, ∆69–70, ∆144, G181V	2 (1.4)
	N501Y, ∆69–70, ∆144, S155R	2 (1.4)
	N501Y, ∆69–70, ∆144, V289L	2 (1.4)
	N501Y, ∆69–70, ∆142, Y144V	2 (1.4)
	N501Y, S98F, ∆144	1 (1)
	N501Y, ∆69–70	1 (1)
	N501Y, ∆69–70, L141F, ∆144	1 (1)
	N501Y, ∆69–70, ∆144, S155R, F374S	1 (1)
	N501Y, A67V, ∆69–70, ∆144	1 (1)
B.1.351/Beta		7 (3)
	D80A, D215G, ∆241–243, K417N, E484K, N501Y	7 (100)
B.1.617.2/Delta		10 (5)
	T95I, G142D, ∆156–157, R158G, L452R, T478K	3 (30)
	G142D, ∆156–157, R158G, L452R, T478K	1 (10)
	G142D, ∆156–157, R158G, A222V, L452R, T478K	1 (10)
	G142D, ∆156–157, R158G, N440T, L452R	1 (10)
	L452R, N501Y	1 (10)
	T478K	2 (20)
	A222V	1 (10)
B.1.525/Eta		1 (1)
	A67V, ∆69–70, ∆144, E484K	1 (100)
Wild type	No mutation	59 (27)
Total		211 (100)

**Table 2:** Susceptibility rate of SARS-CoV-2 variants to SARS-CoV-2 monoclonal antibodies.

SAR-CoV-2 Lineage/WHO Label	Bamlanivimab ^b^	Etesevimab ^b^	Bamlanivimab plus Etesevimab ^a,b^	Casirivimab ^b^	Imdevimab ^b^	Casirivimab plus Imdevimab ^a,b^	Sotrovimab ^b^	Cligavimab ^c^	Tixagevimab ^c^	Cligavimab plus Tixagevimab ^a^	C135 ^d^	C144 ^d^	C135 Plus C144 ^a^	BRII-196 ^d^	BRII-198 ^d^	BRII-196 plus BRII-198 ^a^
B.1.1.7/Alpha																
Susceptible	133 (99)	1 (1)	131 (98)	134 (100)	134 (100)	119 (89)	23 (17)	134 (100)	134 (100)	133 (99)	120 (90)	6 (4)	6 (4)	120 (90)	120 (90)	113 (84)
Intermediate	ND	22 (16)	ND	ND	ND	1 (1)	111 (83)	ND	ND	ND	ND	ND	ND	ND	ND	ND
Resistance	1 (1)	111 (83)	ND	ND	ND	ND	ND	ND	ND	ND	ND	ND	ND	ND	ND	ND
No data available	-	-	3 (2)	-	-	14 (10)	-	-	-	1 (1)	14 (10)	128 (96)	128 (96)	14 (10)	14 (10)	21 (16)
B.1.351/Beta																
Susceptible	-	-	-	-	-	-	-	-	-	-	-	-	-	-	-	-
Intermediate	-	-	-	-	-	-	-	-	-	-	-	-	-	-	-	-
Resistance	-	7 (100)	-	-	-	-	-	-	-	-	-	-	-	-	-	-
No data available	-	-	-	-	-	-	-	-	-	-	-	-	-	-	-	-
B.1.617/Delta																
Susceptible	2 (20)	7 (70)	ND	5 (50)	9 (90)	8 (80)	1 (10)	-	-	-	-	3 (30)	3 (30)	1 (10)	-	-
Intermediate	ND	ND	1 (10)	4 (40)	ND	1 (10)	ND	-	-	-	-	ND	ND	ND	-	-
Resistance	6 (60)	1 (10)	ND	ND	ND	ND	ND	-	-	-	-	ND	ND	ND	-	-
No data available	2 (20)	2 (20)	9 (90)	1 (10)	1 (10)	1 (10)	9 (90)	-	-	-	-	7 (70)	7 (70)	9 (90)	-	-

Abbreviations: BAM: Bamlanivimab; ETE: Etesevimab; CAS: Casirivimab; IMD: Imdevimab; SOT: Sotrovimab; CIL: Cligavimab; TIX: Tixagevimab; -: no data available; ND: not determined. ^a^ Monoclonal antibody cocktails; ^b^ Emergency use authorized; ^c^ Completed phase III; ^d^ Phase III [26,27].

**Table 3:** Susceptibility rate to convalescent plasma in the determined SARS-CoV-2 variants.

SARS-CoV-2 Variant	Convalescent Plasma Susceptibility, n (%)			
	Susceptible	Intermediate	Resistance	No Data
B.1.117/Alpha, n = 134				
N501Y, ∆69–70, ∆144	105 (100)	-	-	-
N501Y, ∆144	7 (100)	-	-	-
N501Y	7 (100)	-	-	-
N501Y, ∆69–70, S98F, ∆144	2 (100)	-	-	-
N501Y, ∆69–70, ∆144, G181V	2 (100)	-	-	-
N501Y, ∆69–70, ∆144, S155R	2 (100)	-	-	-
N501Y, ∆69–70, ∆144, V289L	2 (100)	-	-	-
N501Y, ∆69–70, ∆142, Y144V	2 (100)	-	-	-
N501Y, S98F, ∆144	1 (100)	-	-	-
N501Y, ∆69–70	1 (100)	-	-	-
N501Y, ∆69–70, L141F, ∆144	1 (100)	-	-	-
N501Y, ∆69–70, ∆144, S155R, F374S	1 (100)	-	-	-
N501Y, A67V, ∆69–70, ∆144	1 (100)	-	-	-
B.1.351/Beta, n = 7				
D80A, D215G, ∆241–243, K417N, E484K, N501Y	-	7 (100)	-	-
B.1.617.2/Delta, n = 10				
T95I, G142D, ∆156–157, R158G, L452R, T478K G142D, ∆156–157, R158G, A222V, L452R, T478K G142D, ∆156–157, R158G, N440T, L452R, T478K G142D, ∆156–157, R158G, L452R, T478K L452R, N501Y T478K A222V	- - - 1 (100) 1 (100) 2 (100) 1 (100)	3 (100) 1 (100) 1 (100)	- - -	- - -
B.1.525/Eta, n = 1				
A67V, ∆69–70, ∆144, E484K	1 (100)	-	-	-

**Table 4:** Susceptibility rate of SARS-CoV-2 lineages to all approved COVID-19 vaccines.

	mRNA Vaccine		Viral Vector Vaccine				Inactivated Vaccine			Recombinant Vaccine		Combined Vaccine
SARS-CoV-2 Lineage/WHO Label	Comirnaty (Pfizer-BioNTech)^a^	Moderna ^b^	AstraZeneca ^b^	Sputnik V ^d^	Janssen ^b^	Bharat Biotech ^e^	Sinopharm ^c^	Covaxin ^e^	Corona Vac ^c^	Novovax ^b^	Medigen	Comirnaty (Pfizer-BioNTech) + AstraZeneca
B.1.1.7/Alpha												
Susceptible	134 (100)	134 (100)	ND	114 (85)	114 (85)	114 (85)	114 (85)	114 (85)	114 (85)	114 (85)	114 (85)	ND
Intermediate	ND	ND	114 (85)	ND	ND	ND	ND	ND	ND	ND	ND	114 (85)
Resistance	ND	ND	ND	ND	ND	ND	ND	ND	ND	ND	ND	ND
No data available	-	-	20 (15)	20 (15)	20 (15)	20 (15)	20 (15)	20 (15)	20 (15)	20 (15)	20 (15)	20 (15)
B.1.351/Beta			-	-	-	-	-	-	-		-	-
Susceptible	ND	ND								None		
Intermediate	5 (71)	2 (29)								1 (14)		
Resistance	ND	ND								ND		
No data available	2 (29)	5 (71)								6 (86)		
B.1.617/Delta			-	-	-	-	-	-	-	-	-	-
Susceptible	7 (70)	7 (70)										
Intermediate	1 (10)	ND										
Resistance	ND	ND										
No data available	2 (20)	3 (30)										

Abbreviations: -: no data available; ND: not determined; BNT 162b2: BioNTech, Pfizer vaccine; m RNA 1273: Moderna vaccine; AZD1222: Oxford, AstraZeneca; AD26.COV2. S: Janssen; BBV154: Bharat Biotech; BBIBP-CorV: Sinopharm BIBP COVID-19 vaccine; BBV152: Covaxin COVID-19 vaccine; Corona Vac: SinoVac—CoronaVac vaccine; NVX-CoV2373: Novovax COVID-19 vaccine; MVC-COV1901: Medigen COVID-19 vaccine. ^a^ Food and Drug Administration-approved (FDA-approved) [30] ^b^ Emergency use authorization ^c^ National Medical Products Administration (NMPA) ^d^ Russian National Rifle Association (NRA) ^e^ Drug Controller General of India (DCGI).

## Data Availability

The data presented in this study are available on request from the corresponding author.
